# Protective role of mirtazapine in adult female *Mecp2*^+/−^ mice and patients with Rett syndrome

**DOI:** 10.1186/s11689-020-09328-z

**Published:** 2020-09-28

**Authors:** Javier Flores Gutiérrez, Claudio De Felice, Giulia Natali, Silvia Leoncini, Cinzia Signorini, Joussef Hayek, Enrico Tongiorgi

**Affiliations:** 1 Department of Life Sciences, University of Trieste, Via Licio Giorgieri, 5 - 34127, Trieste, Italy; 2 Neonatal Intensive Care Unit, Azienda Ospedaliera Universitaria Senese, 53100 Siena, Italy; 3 Child Neuropsychiatry Unit, Azienda Ospedaliera Universitaria Senese, 53100 Siena, Italy; 4 Department of Molecular and Developmental Medicine, University of Siena, 53100 Siena, Italy; 5 Pediatric Speciality Center “L’Isola di Bau”, 50052 Certaldo, Florence, Italy

**Keywords:** Rett syndrome, Intellectual disability disorders, Irritability/aggressiveness, Motor learning deficits, Antidepressants, Somatosensory cortex, Parvalbumin neurons

## Abstract

**Background:**

Rett syndrome (RTT), an X-linked neurodevelopmental rare disease mainly caused by *MECP2*-gene mutations, is a prototypic intellectual disability disorder. Reversibility of RTT-like phenotypes in an adult mouse model lacking the *Mecp2-*gene has given hope of treating the disease at any age. However, adult RTT patients still urge for new treatments. Given the relationship between RTT and monoamine deficiency, we investigated mirtazapine (MTZ), a noradrenergic and specific-serotonergic antidepressant, as a potential treatment.

**Methods:**

Adult heterozygous-*Mecp2* (HET) female mice (6-months old) were treated for 30 days with 10 mg/kg MTZ and assessed for general health, motor skills, motor learning, and anxiety. Motor cortex, somatosensory cortex, and amygdala were analyzed for parvalbumin expression. Eighty RTT adult female patients harboring a pathogenic *MECP2* mutation were randomly assigned to treatment to MTZ for insomnia and mood disorders (mean age = 23.1 ± 7.5 years, range = 16–47 years; mean MTZ-treatment duration = 1.64 ± 1.0 years, range = 0.08–5.0 years). Rett clinical severity scale (RCSS) and motor behavior assessment scale (MBAS) were retrospectively analyzed.

**Results:**

In HET mice, MTZ preserved motor learning from deterioration and normalized parvalbumin levels in the primary motor cortex. Moreover, MTZ rescued the aberrant open-arm preference behavior observed in HET mice in the elevated plus-maze (EPM) and normalized parvalbumin expression in the barrel cortex. Since whisker clipping also abolished the EPM-related phenotype, we propose it is due to sensory hypersensitivity. In patients, MTZ slowed disease progression or induced significant improvements for 10/16 MBAS-items of the M1 social behavior area: 4/7 items of the M2 oro-facial/respiratory area and 8/14 items of the M3 motor/physical signs area.

**Conclusions:**

This study provides the first evidence that long-term treatment of adult female heterozygous *Mecp2*^*tm1.1Bird*^ mice and adult Rett patients with the antidepressant mirtazapine is well tolerated and that it protects from disease progression and improves motor, sensory, and behavioral symptoms.

## Background

Rett syndrome (RTT, OMIM #312750) [1, 2] is a postnatal, progressive, non-degenerative neurodevelopmental disorder [3] with an incidence of 1/10,000 female live births [4]. Typical RTT cases arise from de novo mutations in the X-linked gene *MECP2* (methyl-CpG binding protein 2, HGNC:6990) [5], a context-dependent global organizer of chromatin architecture regulating transcription of numerous genes [6, 7]. Heterogeneity in *MECP2* gene mutations [8] and cellular mosaicism derived from random X-chromosome inactivation (XCI [9];) contribute to highly variable symptomatology [10]. However, four symptoms are common to all typical RTT patients: loss of hand skills, loss of spoken language, gait abnormality, and stereotypic hand movements [11]. RTT clinical progression can be divided into four stages: a developmental stagnation stage, a rapid regression stage with loss of previously learned skills, a stationary stage, and a late motor deterioration stage in adulthood [12]. The latter usually leads to parkinsonism [13], but it often includes an improvement in social communication skills [14]. Because RTT first appears in childhood, most studies have focused on treatments at an early age, while studies on adult RTT are comparatively fewer. However, as relevant advances in medicine have allowed extending life expectancy in women with RTT [15], there is currently a strong need to find pharmacological treatments able to increase the quality of life of adult RTT people.

The demonstration that several RTT neurological defects can be rectified by re-expressing *Mecp2* gene in adult mice [16], together with the lack of neuronal loss [17, 18], indicated that RTT is not an irrevocable disease. However, approaches aiming to restore the normal gene dosage are far from being achieved [19], and the only available therapies for RTT are symptomatic, especially to limit seizures [20]. Based on the observation that the monoamines serotonin, noradrenaline, and dopamine are reduced in RTT patients and mouse models [21–23], antidepressants have emerged as potential treatments for RTT. Indeed, desipramine, a noradrenaline-reuptake inhibitor, was successfully tested in mouse models commonly used to study RTT [24, 25] but did not show clinical efficacy in a phase-II clinical trial including 36 RTT girls and presented some relevant side effects [26]. Nonetheless, these studies provided relevant reasons to further investigate the noradrenergic pathway. Accordingly, we hypothesized to use mirtazapine (MTZ), a widely used noradrenergic and specific-serotonergic antidepressant (NaSSA) that has an excellent safety profile [27], as it lacks anticholinergic [28] and cardiorespiratory side effects [29]. We previously tested MTZ in male *Mecp2*^tm1.1Bird^ null mice [20, 30], observing the rescue of several behavioral, physiological, and neuronal morphology phenotypes after only 2 weeks of treatment [31]. However, the male *Mecp2*^y/−^ knockout mouse model misses several aspects of the disease which instead are present in female mice, such as heterozygosity of *Mecp2* mutations due to XCI and a longer lifespan, which allows studies in adulthood [30]. Accordingly, here we investigated the effects of a long-term MTZ treatment in adult female heterozygous (HET) *Mecp2*^tm1.1Bird^ mice and adult female RTT patients.

## Methods

### Animals

Animals were treated according to the institutional guidelines, in compliance with the European Community Council Directive 2010/63/UE for care and use of experimental animals. Authorization for animal experimentation was obtained from the Italian Ministry of Health (Nr. 124/2018-PR, with integration Nr. 2849TON17 for whisker clipping), in compliance with the Italian law D. Lgs.116/92 and the L. 96/2013, art. 13. All efforts were made to minimize animal suffering and to reduce the number of animals used. For animal production, *Mecp2* HET females (*Mecp2*^+/−^, B6.129P2(C)-*Mecp2*^tm1.1Bird/J^, Stock No: 003890. The Jackson Laboratory, USA [30]) were bred with wild-type C57/BL6J male mice (The Jackson Laboratory, USA). We used female *Mecp2*^*+/−*^ mice at 6 months of age, when they present several consistent RTT-like phenotypes [30] and represent, therefore, the most reliable mouse model to study RTT. After weaning, wild-type (WT) and HET mice were housed in ventilated cages under 12 h light/dark cycle with food and water ad libitum. No environmental enrichment elements were added to the cages. All experiments were performed blind to the genotype and treatment of animals, and all control animals were WT age-matched littermates of HET mice. Finally, mice were assigned to groups according to the rules indicated by [32].

### Mice genotyping

Biopsies from ear punches were incubated with 250 μL of DNA extraction buffer (TRIS 10 mM pH 7.5, EDTA 5 mM pH 8, SDS 0.2%, NaCl 10 mM, proteinase K 0.5 mg/mL) and left overnight at 55 °C. The day after, samples were centrifuged (12000 rpm, 20 min, RT), then 100 μL of the supernatant were mixed with isopropanol (1:1), and precipitated DNA was centrifuged again (12000 rpm, 30 min, RT). Supernatant was then discarded, and three washes with cold 70% ethanol with subsequent centrifugations (12000 rpm, 5 min, RT) were realized. Once ethanol had evaporated, DNA pellets were homogeneously dissolved in milli-Q water. Genotypes were assessed by PCR on genomic DNA extracted from ear-clip biopsies. PCR reactions were performed using specific primers (forward common primer oIMR1436 5′-GGT AAA GAC CCA TGT GAC CC-3′, reverse mutant primer oIMR1437 5′-TCC ACC TAG CCT GCC TGT AC-3′) with 1 U GoTaq polymerase (Promega, Madison, USA), 1X green GoTaq buffer, 0.2 mM dNTPs each, 2.5 mM MgCl_2_, 0.5 μM of each primer, and 10 ng/μL of genomic DNA, as follows: 95 °C, 3’ > 30 cycles: 95 °C, 20”; 58 °C, 20”; 72 °C, 20” > 72 °C, 2’. This PCR generates a 400-bp product for WT allele and an additional 416-bp product for heterozygous mice [31].

### Mice treatment

Beginning from 5 months of age, HET females and WT littermates were i.p. injected with vehicle (VEH = 0.9% aqueous solution of NaCl and 5% ethanol) or MTZ (10 mg/kg, ab120068, Abcam, Cambridge, UK). The 10 mg/kg daily dosage in mice is equivalent to 50 mg/day in humans, which is the maximum dose used in patients [33]. To calculate it, we followed a dose by factor method modified from [34]. The next formula was used:

$$ \mathrm{Animal}\ \mathrm{dose}\ \left(\mathrm{mg}/\mathrm{kg}\right)=\frac{\mathrm{human}\ \mathrm{dose}\ \left(\mathrm{mg}/\mathrm{kg}\right)}{{\left[\mathrm{weight}\ \mathrm{mouse}\ \left(\mathrm{kg}\right)/\mathrm{weight}\ \mathrm{human}\left(\mathrm{kg}\right)\right]}^{0.33}} $$

We used standard weights described by [34] (mouse = 0.02 kg; human 60 kg), obtaining a dose of 11.7 mg/kg, which we rounded to 10 mg/kg.

As the half-life of MTZ is about 48 h, mice were treated on alternate days, for 30 days, at 10–11 a.m. For the randomization of groups, we first realized a general phenotypic scoring [16] of all WT and HET mice. Based on the results of this evaluation, we homogeneously divided WT and HET mice to create our four experimental groups: WT-VEH, WT-MTZ, HET-VEH, and HET-MTZ.

### Whisker clipping

This procedure was applied only to a group of untreated WT and HET mice. Before the whisker clipping, mice were injected with 100 μg/kg medetomidine (Domitor®, Vetoquinol, Magny-Vernois, France), an α-2 adrenergic agonist with hypnotic and sedative effects, to prevent potentially risky movements. We then left mice into an incubator at 37 °C, and when they were completely sedated, we proceeded to shorten whiskers up to a few millimeters from the skin (leaving untouched whisker bulbs). Immediately after that, unwhiskered mice were i.p. injected with 1 mg/kg atipamezole (Antisedan®, Zoetis, New Jersey, USA), a specific antagonist of α-2 adrenergic receptors. Mice were then kept for some hours in an incubator at 30 °C. When they were completely awake, we put them back in their home cages and waited until the day after to perform the elevated plus maze.

### Animal behavior

**Fig. 1:**
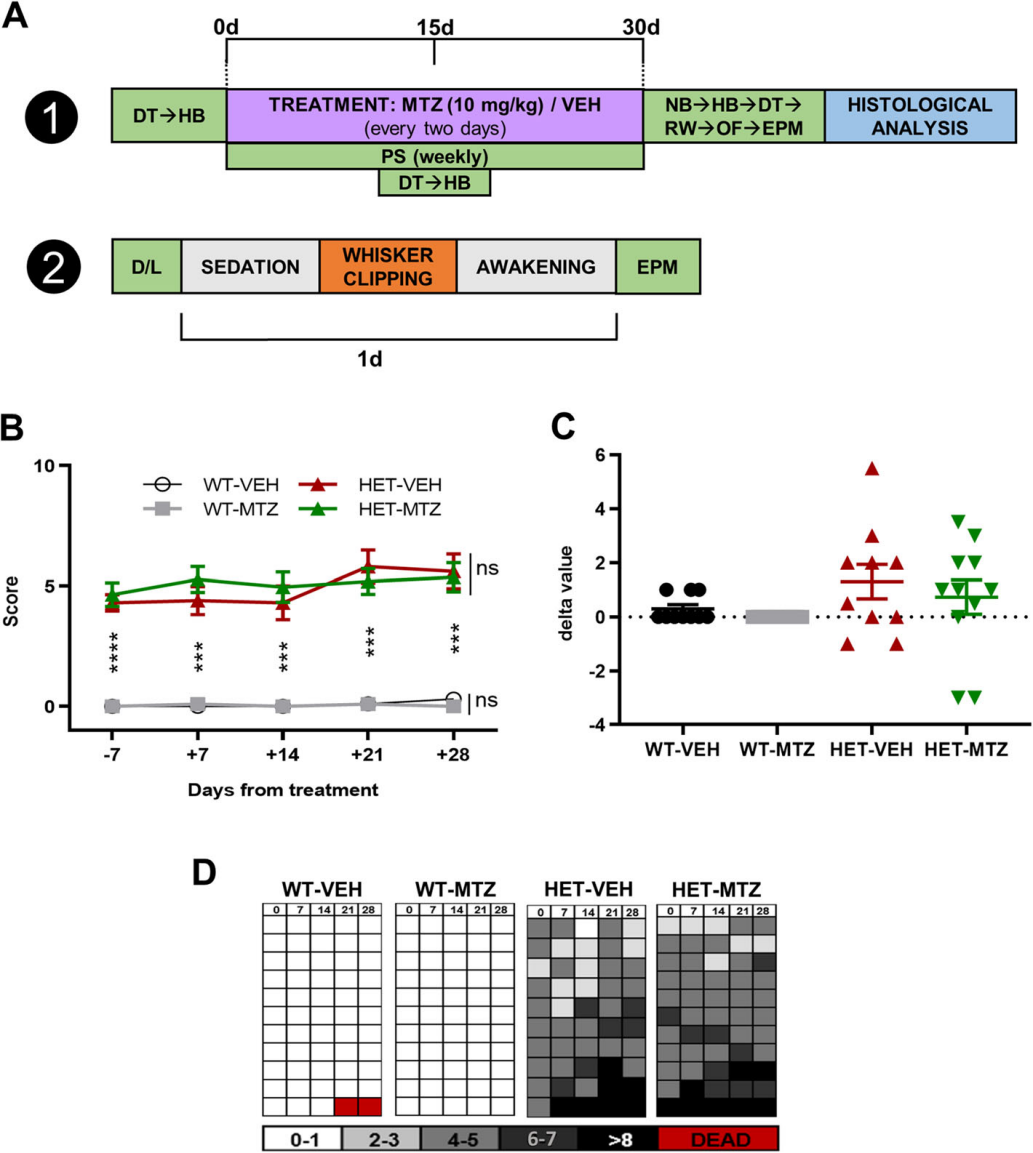
General health evaluation of 6-month-old WT and HET *Mecp2*^tm1.1Bird^ female mice before, during, and after treatment with MTZ (10 mg/kg). **a** Two protocols are shown. In the first, the main experimental timeline is detailed. Before the treatment, we evaluated general health through a specific phenotypic scoring (PS), besides dowel (DT) and horizontal bar (HB) tests. During the treatment phase (30 days), we performed, on alternate days, either i.p. injections of 10 mg/kg MTZ/vehicle (VEH) or behavioural tests: PS, DT, HB, and the nest building test (NB). Within the post-treatment phase (the week following the last injection), we performed DT, rod walk (RD), open-field (OF), and elevated plus maze (EPM) tests. At the end of this phase, mice brains were dissected and prepared for histological analysis. The second protocol was used only on untreated 6-month-old WT and HET mice. These mice were first tested at the dark-light exploration test (D/L). The day after, they were sedated, and their whiskers were clipped. They were then left in their home cages overnight, and the day after they were tested at the EPM. **b** Main evaluation of the phenotypic scoring along the experimental timeline. **c** Delta values for the main evaluation of the PS, calculated as the difference between the last evaluation (post-treatment) and the first one (pre-treatment). **d** Grid representing time course of PS for each single mouse. Within each experimental group, each row represents a different mouse. Data on graphs are expressed as median ± interquartile range, *n* = 10–11 mice per group. According to results of Shapiro-Wilk test, we performed Kruskal-Wallis test followed by corrected Dunn’s post hoc test. Multiple selected comparisons comprehended: WT-VEH vs WT-MTZ, WT-VEH vs HET-VEH, and HET-VEH vs HET-MTZ. ns: *p* > 0.05 (not shown); *p ≤ 0.05; **p ≤ 0.01; ***p ≤ 0.001

General scheduling of treatment and behavioral testing is presented in Fig. 1a. In short, we first performed a general phenotypic scoring and two motor tests before and during the treatment, to see individual and fast effects of MTZ. At the end of the treatment, we realized further tests for a better assessment of treatment effects. In addition, we realized a separated behavioral assessment of untreated mice where whiskers had been cut.

### Phenotypic scoring

Mice were removed from their home cages and place onto a laboratory bench for observation at the same time of the day. Phenotype severity was evaluated through a scoring system modified from [16]. The main evaluation comprehends the following features: mobility, gait, hindlimb clasping, tremor, breathing, and state of fur and eyes. Scoring was as follows: 0 = absent or as observed in WT; 1 = moderate phenotype; 2 = severe phenotype. By summing these values, we obtained an average value for each mouse, being 12 points the maximum possible scoring. We also evaluated descriptively the following features: presence of reactive vocalizations, eyes aperture, hierarchy inside the cage based on loss of hair in the back due to barbering behavior, presence of involuntary (clonic and tonic) movements, and grooming during observation. All treated mice (*n* = 10–11 per group) where analyzed through this scoring.

### Dowel test

Following a modified protocol based on [35], we positioned the mouse with its four paws on the free edge of a striped 60-cm long and 10-mm thick wooden dowel and measured the latency to fall (endpoint = 120 s). The test was repeated before, during, and after the treatment. All treated mice (*n* = 10–11 per group) were analyzed through this test.

### Horizontal bar test

Following a modified protocol based on [35], we let the mouse hang, with his forelimbs only, to a horizontal bar and then we measured the latency to fall (endpoint = 30 s). An individual score was assigned to each mouse: 1–5 s = 1 point; 6–10 s = 2 points; 11–20 s = 3 points; 21–30 s = 4 points; no fall = 5 points; travelling to the end of the bar = 6 points. The test was consecutively performed on bars with decreasing diameters (4 and 2 mm). Corresponding scores from both bars were summed, obtaining an individual score for each mouse. All treated mice (*n* = 10–11 per group) were analyzed through this test.

### Nest building test

Following a protocol that takes advantage of the natural inclination of mice to create a nest with different materials [36], we put mice in isolated cages 1 h before the beginning of the dark phase. We kept them in the individual cages for 16 h, in the presence of a 3-g Nestlet (Ancare, New York, USA) but without any other environmental enrichment. The day after, mice were returned to their home cages, nests were visually assessed, and remaining pieces of the Nestlet were weighed. All treated mice (*n* = 10–11 per group) were analyzed through this test.

### Rod walk test

Following a modified protocol based on [35], we positioned the mouse at one edge of a 60-cm wood dowel (standing 50 cm above the floor), where a repulsing stimulus (strong light) was present. On the other side of the dowel, we positioned an attractive stimulus (dark cage with nesting material from the mouse’s home cage). Transition time was measured in two consecutive trials, performed with striped dowels of 12 and 10 mm of diameter. All treated mice (*n* = 10–11 per group) were analyzed through this test.

### Open field

Each mouse was individually placed in the center of the open field (40 × 40 × 40 cm) and left to freely explore it for 20 min. Movements of mice were recorded from above to avoid interference of the experimenter, and videos were later analyzed with the ANY-maze software (Stoelting, New Jersey, USA), as previously described [31]. In short, we divided the open field area into central, middle, and border zones and measured automatically or manually the following parameters: entries and time spent in each zone, total traveled distance, mean speed, immobility episodes, grooming levels, vertical activity, and hopping behavior. All treated mice (*n* = 10–11 per group) were analyzed through this test.

### Elevated plus maze

Each mouse was individually placed in the center area of a black plexiglass elevated plus maze for a 5-min test session [37]. Mice movements were recorded and later analyzed with the ANY-maze software (Stoelting, New Jersey, USA) to automatically measure entries and time spent in the open and in the closed arms. All treated mice (*n* = 10–11 per group) and untreated unwhiskered mice (*n* = 7 per group) were analyzed through this test.

### Dark-light exploration

This test was performed as previously described [37]. The apparatus was made of two plexiglass compartments separated by a partition with a small opening. One compartment was transparent and illuminated, while the other was opaque and closed on top. Each mouse was individually placed into the center of the light compartment and allowed to freely explore both areas for 10 min. We video-tracked the movements of mice and then measured the number of transitions between light and dark sides, as well as time spent in each compartment, by using the ANY-maze software (Stoelting, New Jersey, USA). Only untreated unwhiskered WT and HET mice (*n* = 7 per group) were analyzed through this test.

### Histological analyses

All mice were sacrificed at the end of behavioral testing (Fig. 1a), and dissected brains were fixed on PFA 4% solution (24 h, 4 °C). They were then washed and cryoprotected in an increasingly concentrated sucrose solution (20% and 30%) and stored at 4 °C. We produced 20-micron transversal sections with a cryostat (Leica, Wetzlar, Germany) from approximately Bregma +1.54 to Bregma −1.58 [38]. This segment includes our three regions of interest: the primary motor cortex (M1), the primary somatosensory-barrel cortex (S1), and the baso-lateral amygdala (BLA).

### Analysis of cortical thickness

PFA-fixed slices from treated mice brains were stained in cresyl violet solution (0.2% cresyl violet (Sigma), 0.5% glacial acetic acid, 0.01 M sodium acetate) for 20 min at 37 °C. Sections were then sequentially dehydrated with rapid washes in: 70% ethanol, 95% ethanol, 100% ethanol, methanol, 1:1 methanol-xylene. Finally, glasses were covered by using Eukitt (Sigma, Saint Louis, USA). Pictures of Nissl-stained brain sections were acquired with a Nikon AMX1200 digital camera on a Nikon E800 Microscope (× 4 magnification). Bregma position was assigned to each slice following [38] and then the FIJI software was used to measure cortical thickness [31]. First, we produced a densitometrical plot profile from a line spanning the cortex perpendicularly from the pial surface to the white matter underlying cortical layer VI. We then identified transitions among layers as variations in the densitometrical plot profile, which reflect cell density changes between cortical layers, and measured their lengths in the plot (see Supplementary Fig. S3). This approach allowed us to measure the total cortical thickness in S1 and M1, and the relative thickness of cortical layers I, II/III–IV, and V–VI in the S1. Eight mice per group and 10–15 slices per mice were analyzed.

### Immunofluorescence

Free-floating slices were permeabilized (1% Triton X-100) for 1 h at RT, then incubated in blocking solution (0.1% Triton X-100, 2% BSA) for 1 h at RT, and finally incubated in fresh blocking solution containing 1:5000 anti-parvalbumin antibodies (PV235, Swant) overnight at 4 °C. The day after, slices were washed and then incubated in PBS containing 1:250 Alexa Fluor 568 donkey anti-rabbit antibodies (A-10042, Life Technologies). Slices were finally incubated in 1:1000 Hoechst solution (33342, Sigma).

### Analysis of parvalbumin-positive (PV+) cells

Fluorescence images were acquired with a Nikon Eclipse Ti-E epifluorescence microscope equipped with a Nikon DS-Qi2 camera. We first used a × 4 magnification to identify the Bregma position of each slice and then took a large picture with a × 10 magnification (2 × 2 fields, total area = 1,310,720 μm^2^) of the correspondent regions of interest (Supplementary Fig. S2). Acquisition and analyses were performed by using the NIS-Elements software (v4.60. Nikon). We generated digital boxes (width = 230.34 μm) spanning from the pial surface to the corpus callosum that were superimposed at the level of the barrel and primary motor cortices on each brain slice. PV+ and Hoechst-positive cells were counted automatically after having applied an automatic intensity threshold specific for each picture. From 5 to 10 slices for each mouse, and 4–5 mice per group were analyzed. We used the FIJI software [39] to measure the intensity of PV+ cells. In short, we manually created specific ROIs for PV+ cells somata and then automatically measured their mean pixel intensity. Three additional ROIs were used to measure the background for each picture, which was later subtracted from the mean intensity in the correspondent PV+ cells. Since PV+ positive cells showed a high signal-to-noise ratio after background subtraction, no further post-processing image manipulation was carried out. Between 177 and 283 neurons for each experimental group (for cortical ROIs) and between 38 and 88 neurons for each experimental group (for BLA) were evaluated through this protocol. For histological analyses, each experimental group was composed of 4–5 mice with average levels in behavioral phenotypes.

### Study design and patients

This is a retrospective study on 80 adult female patients diagnosed with Rett syndrome. All clinical examinations were performed at the Child Neuropsychiatry Unit, University Hospital Le Scotte, Siena, Italy, between the years 2012 and 2019. Clinical phenotype was compatible with typical RTT for 76 patients (95% of total sample) while 4 presented with preserved speech variant (PSV) [40]. Forty RTT adult female patients harboring a pathogenic *MECP2* mutation (37 typical and 3 PSV patients) were treated with MTZ for insomnia and mood disorders (mean age = 23.9 ± 7.9 years, range = 16–47 years; mean MTZ-treatment duration = 1.64 ± 1.0 years, range = 0.08–5.0 years). For comparative purposes, a genetically heterogeneous, age-matched cohort of 40 RTT patients (39 typical and 1 PSV patient) not receiving MTZ was considered as the control group. Informed consent to participate in the study was obtained by the parents/caregivers. Ethical approval was waived by the local Ethics Committee of Azienda Sanitaria of Siena in view of the retrospective nature of the study and all the procedures being performed were part of the routine care. The retrospective investigation was conducted according to the Ethics Guidelines of the institute and the recommendations of the declaration of Helsinki and the Italian DL-No.675/31-12-1996.

### Clinical data

Demographic data, type of *MECP2* mutation, MTZ dosage, duration of treatment, and clinical severity at baseline as evaluated by Rett syndrome clinical severity scale were collected (Tables 1 and 2). The RCSS, a validated RTT-specific scale designed to assess the severity of key symptoms, was completed by the same clinician at each visit. It consists of 13 items providing a rating of core symptoms of RTT on a Likert scale of either 0 to 4 or 0 to 5 with a maximum total score of 58. Efficacy was gauged through possible variations in illness severity as a function of MTZ treatment based on variations in motor behavioral assessment scale (MBAS) score. The MBAS includes a subset of 37 items, each one ranging 0 to 4, with a total sum ranging 0 to 68. Both scores are known to be proportional to illness severity. Possible changes in the MBAS item subscores were considered. Both RCSS and MBAS were rated by experienced clinicians (JH, CDF). To reduce inter-observer variability, the average score between the two physicians was used. MTZ safety and tolerability were monitored by clinicians during the scheduled visits at the center and through periodic phone contacts with parents. Drug tolerance and the occurrence of possible adverse events were also recorded.

**Table 1:** Demographics and relevant features of untreated RTT vs patients treated with mirtazapine (MTZ)

Variable	Untreated RTT	MTZ-treated RTT	*p* value
*N*	40	40	
Age (years)	22.3 ± 7.2	23.9 ± 7.9	0.354
Clinical phenotype			0.079
Typical	39 (97.5%)	37 (92.5%)	
Preserved speech variant (PSV)	1 (2.5%)	3 (7.5%)	
*MECP2* mutation category			0.223
Missense	17 (42.5%)	13 (32.5%)	
Early truncating	15 (37.5%)	17 (42.5%)	
Late truncating	4 (10%)	9 (22.5%)	
Gene deletions	4 (10%)	1 (2.5%)	
Rett syndrome clinical severity scale (RCSS)	24.7 ± 8.2	22.6 ± 6.5	0.206
Motor behavioral assessment scoring scale (MBAS)	56.4 ± 9.0	58.7 ± 6.5	0.596
I Social-behavioral domain	22.7 ± 6.1	24.0 ± 6.9	0.374
II Orofacial/respiratory domain	13.6 ± 2.8	14.2 ± 3.9	0.417
III Motor and clinical features domain	20.1 ± 3.3	20.5 ± 1.0	0.450

Patient’s diagnosis is indicated as typical or preserved speech variant (PSV). Symptomatic severity was assessed through the Rett syndrome clinical severity scale (RCSS) and motor behavioral assessment scoring scale (MBAS). All patients were females. Data are expressed as mean ± standard deviation or *N* (percentages)

**Table 2:** Dosages and duration of Mirtazapine (MTZ) treatment on patients

MTZ treatment in RTT				
Patient	Daily dose (mg/day)	Per kg dose (mg kg bw/day)	Treatment duration (years)	Tolerance
No. 1	7.5	0.170	2	T
No. 2	15	0.375	2	T
No. 3	7.5	1.071	2	T
No. 4	30	0.428	5	T
No. 5	30	0.806	2	T
No. 6	15	0.395	1	T
No. 7	15	0.288	2	NT
No. 8	22.5	0.625	3	T
No. 9	7.5	0.306	1	T
No. 10	15	0.288	2	T
No. 11	15	0.441	0.08	NT
No. 12	3.75	0.122	1.4	T
No. 13	3.75	0.170	1	T
No. 14	7.5	0.250	1.2	T
No. 15	7.5	0.187	0.08	NT
No. 16	15	0.273	0.5	NT
No. 17	7.5	1.970	1.4	T
No. 18	3.75	0.288	1	T
No. 19	7.5	0.115	2	T
No. 20	15	0.300	1.7	T
No. 21	7.5	0.263	1.1	T
No. 22	7.5	0.250	1.4	T
No. 23	7.5	0.166	1.2	T
No. 24	15	0.250	1.5	T
No. 25	15	0.220	1.1	T
No. 26	15	0.211	1	T
No. 27	7.5	0.227	1.2	T
No. 28	15	0.286	1	T
No. 29	15	0.214	2.4	T
No. 30	15	0.300	2.1	T
No. 31	15	0.329	5	T
No. 32	7.5	0.205	2.3	T
No. 33	7.5	0.246	2	T
No. 34	7.5	0.117	1.1	T
No. 35	7.5	0.227	1.5	T
No. 36	3.75	0.174	1	T
No. 37	7.5	0.246	2.2	T
No. 38	7.5	0.258	1.3	T
No. 39	7.5	0.246	2.2	T
No. 40	3.75	0.134	1.2	T

Dosages were personalized for each patient according to clinical observations. Data are expressed as daily dose (mg/day) and per kg dose (mg/kg bw/day) of MTZ. Duration of MTZ treatment was always longer than 1 year, with four exceptions (patients Nos. 7, 11, 15, and 16), in whom an intolerance (NT) to the drug was observed. In all the other cases, MTZ treatment was well tolerated (T)

### Experimental design and statistical analysis

All mouse behavioral experiments were done blindly to the experimenter, and all experimental groups were tested in the same session in a randomized order. As reported in the immunohistological techniques section, we analyzed from 5 to 10 slices for each mouse, in order to have representative single values. For the retrospective analysis of patients treated with MTZ, the sample size was equal to the number of patients treated with MTZ available. For all experiments, we realized a post hoc analysis to determine the power of the analysis by using the G*Power 3 software [41]. We verified that all significant results on which we based our conclusions had an associated power analysis (1-β) higher than 0.8 and, therefore, they can be considered as statistically reliable. Data analysis and data graphics were performed with the GraphPad Prism 7.0 software (GraphPad, La Jolla, California, USA). Parametric data are expressed as mean ± standard deviation (SD) and non-parametric data as median ± interquartile range. To test differences between two groups, Student’s *t* test, Mann-Whitney test, or Wilcoxon signed-rank test were used as appropriate. One-way ANOVA (with the Dunnett’s post hoc test) or Kruskal-Wallis test (with the corrected Dunn’s post hoc multiple comparisons test) were used for comparing multiple groups. The combined effect of two independent variables was tested using two-way ANOVA (with the Dunnett’s post hoc test). The chi-squared test was used for categorical variables in the study of patients. All outliers were detected using Grubb’s test.

## Results

### General health in HET mice is not affected by MTZ

To determine whether a 30-day treatment (through i.p. injections on alternate days) with MTZ at 10 mg/kg (a dose equivalent to the maximum dosage used in humans, 50 mg/day, see details in the “Methods” section) was able to improve general health in adult (6-month-old) HET female mice, we performed a phenotypic analysis using a modified protocol from [16] (see “Methods”) at the end of each week of treatment, including a pre-treatment time point (Fig. 1a). The general health of vehicle-treated HET (HET-VEH) mice was significantly reduced compared to vehicle-treated wild-type (WT-VEH) mice already before the treatment (*p* < 0.0001, corrected Dunn’s post hoc test) and remained unchanged along the analyzed period (Fig. 1b). No significant improvement was observed in HET mice treated with MTZ (HET-MTZ) compared to HET-VEH mice at the end of the treatment (*p* > 0.9999, corrected Dunn’s post hoc test (Fig. 1c). Regarding the possible side effects of MTZ, we verified the complete safety of the treatment, as no phenotypic alteration was observed in WT mice treated with MTZ (WT-MTZ) compared to WT-VEH control mice at the end of the treatment (*p* > 0.9999, corrected Dunn’s post hoc test; Fig. 1b).

### MTZ prevents the progression of motor deficits in adult HET mice

**Fig. 2:**
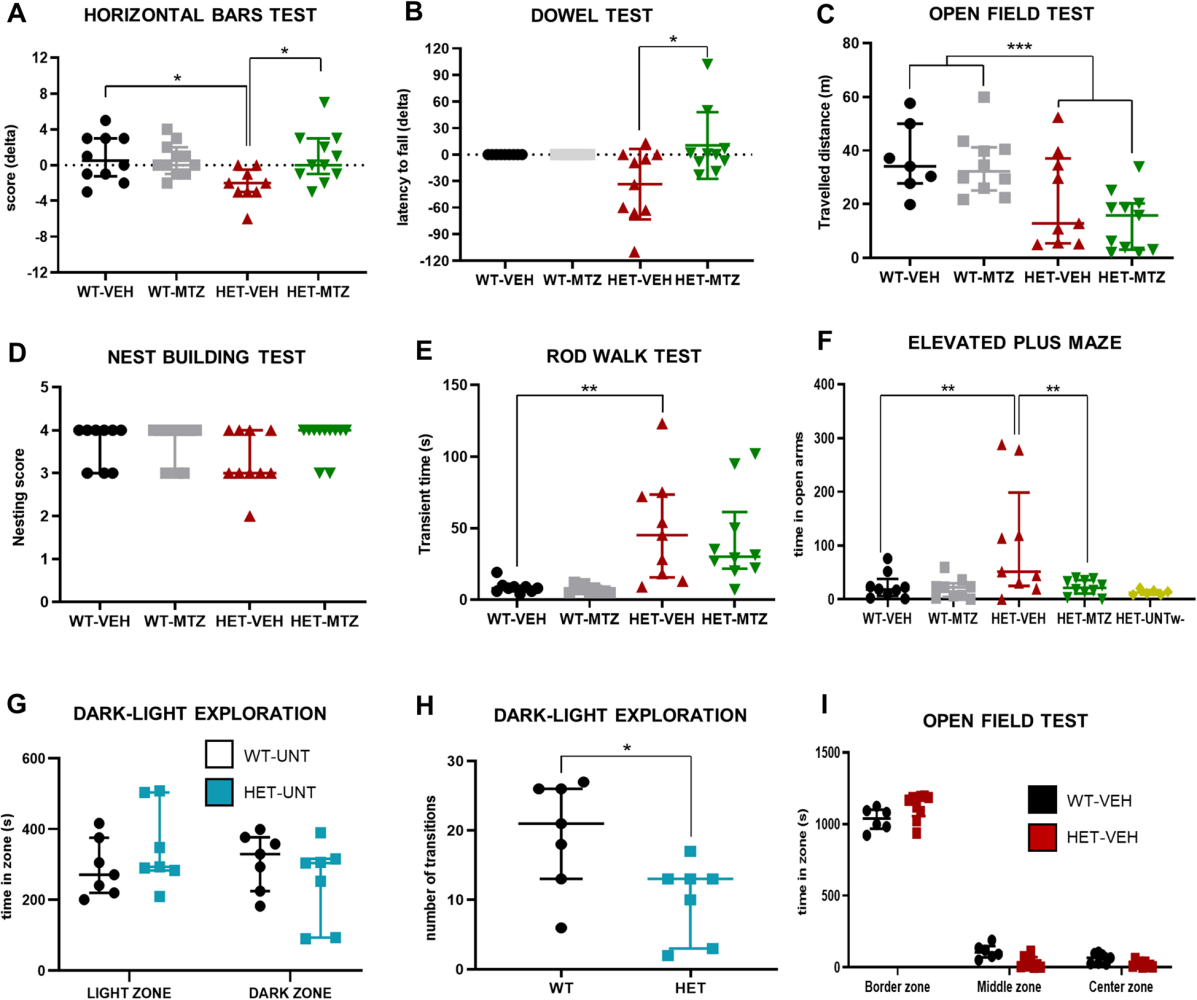
Assessment of motor phenotypes and anxiety-related behaviours of 6-month-old *Mecp2*^tm1.1Bird^ female mice after treatment with MTZ (10 mg/kg). **a** Motor learning at the horizontal bar test, measured as the change (delta value) between performance at the end and at the beginning of the treatment. **b** Motor learning at the dowel test, measured as the change (delta values) between performance at the end and at the beginning of the treatment. **c** Total travelled distance in the open field. **d** Scoring of the nests built by mice after 24 h in isolation. **e** Transient time at the rod walk test. All data are expressed as median ± interquartile range, *n* = 10–11 mice per each group. **f** Time spent in open arms in the elevated plus maze in wild-type (WT) and heterozygous (HET) mice, treated with either mirtazapine (MTZ) or vehicle (VEH). A group of untreated HET mice whose whiskers were clipped (HET-UNTw-) is also included in the graph. **g** Time spent in zones in the dark-light exploration test by untreated (UNT) WT and HET mice. **h** Number of transitions between the light and the dark zones. **i** Time spent in each zone in the open field by WT and HET mice after treatment with VEH. All data are expressed as median ± interquartile range, *n* = 7–11 mice per group. According to results of Saphiro-Wilk test, we performed either one-way ANOVA followed by Sidak’s multiple comparison test or Kruskal-Wallis test, followed by corrected Dunn’s post hoc test. Two-way ANOVA multiple selected comparisons comprehended: WT-VEH vs WT-MTZ, WT-VEH vs HET-VEH, and HET-VEH vs HET-MTZ. In order to determine an eventual genotype effect, 2-way ANOVA was performed in the open-field test. *p ≤ 0.05, **p ≤ 0.01, ***p ≤ 0.001

Progressive motor deficits are a key feature of RTT which has been largely described in patients and in male and female murine models used to study this disease [37, 42]. We evaluated whether MTZ was able to rescue motor phenotypes in adult HET mice by testing motor performance at three different time points: before, during, and after the treatment (Fig. 1a). In the horizontal bar test (HB) both WT-VEH and WT-MTZ mice showed, on average, a better performance at the end compared to the beginning of the testing period, as the mathematical differences (deltas) of scoring between both time points were positive (WT-VEH: delta = 0.80, WT-MTZ: delta = 0.63; Fig. 2a). These results likely reflect the acquisition of learned motor skills upon the repetition of the tests. In the Dowel test using the wooden dowels with a diameter of 10 mm, delta values of latency to fall were equal to zero for every single WT mouse, as all of them reached the endpoint (120 s standing on the dowel) before and after the treatment (Fig. 2b). Instead, HET-VEH mice showed negative delta values in both tests (HB: delta = − 2.22; DT: delta = − 33.4), indicating worsening of the general motor performance (Fig. 2a, b; disaggregated scores in Supplementary Fig. S1A-B, E-F). MTZ treatment significantly prevented HET mice from worsening, as delta values were close to zero in both tests, and even improved performance in half of HET-MTZ mice (Fig. 2a, b). Horizontal bars and dowel tests were also performed in the middle of the treatment to detect possible rapid effects of MTZ, but no significant changes on HET-MTZ mice compared to HET-VEH mice were observed (HB: *p* > 0.9999; DT: *p* = 0.6284; corrected Dunn’s post hoc test), suggesting that long-term treatment is necessary to see effects of MTZ (compare Fig. 2a, b with Supplementary Fig. S1C-D). Of note, the motor deficits and the recovery of the motor performance could only be seen using the 10-mm diameter dowels and not with the 12-mm dowel (Supplementary Fig. S1), likely because the test on the larger wooden rod was less challenging for the HET mice.

In addition to the HB and the DT, three further motor tests were performed only at the end of the treatment. In the open field, used to assess both the general locomotor activity and anxiety levels, HET mice showed a significantly shorter distance traveled during the observation time (20 min) in comparison to WT mice (*F* = 16.33, *p* = 0.0003, two-way ANOVA), and MTZ did not show any significant effect in either genotype (*F* = 1.27, *p* = 0.2686; Fig. 2c). In the nest building test, which evaluates the fine motricity of the forepaws, we did not find any significant phenotype, as no differences were observed between groups (Fig. 2d). In the rod walk test, a clear deficit of general motor coordination was detected (WT-VEH vs HET-VEH: *p* = 0.0068, corrected Dunn’s post hoc test), but no effect of MTZ treatment was observed (HET-VEH vs HET-MTZ: *p* > 0.9999; Fig. 2e). Regarding potential side effects of MTZ in mice, we observed no deleterious effects on motor performance, since the scores of WT-MTZ mice were not significantly different from those of WT-VEH mice for any of the motor tests used. In conclusion, we observed a protective effect of MTZ on motor learning in HET-MTZ mice, with no deleterious effects on motor skills in any used test.

### MTZ rescues the EPM-related phenotype in adult HET mice

Behavioral abnormalities in RTT patients usually include anxiety episodes elicited by distressful external events [43]. In mice, anxiety-related behaviors are typically assessed through the elevated plus maze (EPM), and a characteristic phenotype has been described in both male [31, 42] and young female *Mecp2*^tm1.1Bird^ mice [37]. Specifically, these mice tend to explore longer the open arms compared to their wild-type littermates. We tested 6-month-old female *Mecp2*^tm1.1Bird^ mice in the EPM after a 30-day treatment with MTZ (Figs. 1a, 2f). Our results confirmed the presence of the previously described phenotype (WT-VEH vs HET-VEH: *p* = 0.0095, corrected Dunn’s post hoc test, one-way ANOVA) and demonstrated a complete recovery by MTZ, as HET-MTZ mice explored the maze following the same pattern of WT-VEH mice (Fig. 2f). These results are perfectly in agreement with those we previously obtained in male mice treated with MTZ [31].

### EPM-related phenotype is likely due to enhanced sensitivity of whiskers

According to the standard interpretation, an extended time of exploration of the open arms in the EPM reflects a state of lower anxiety or a high risk-taking behavior. However, this contrasts with the typical symptomatology found in RTT patients who instead present increased levels of anxiety [44]. We then hypothesized that the aberrant behavior of HET mice at the EPM could represent an avoidance of the closed arms due to whisker hypersensitivity, rather than a preference for the open arms. According to this supposition, the narrow, closed arms of the EPM would represent a disturbing stimulus for HET mice. To verify this hypothesis, untreated 6-month-old HET mice were submitted to clipping of all the whiskers (HET-UNTw^-^) and on the day after were tested at the EPM. We observed that the open-arm preference phenotype described before was completely abolished in HET-UNTw^-^ mice (Fig. 2f). To corroborate these results, we investigated if any anxiety-related phenotype was present in adult female *Mecp2*^tm1.1Bird^ mice. Firstly, we tested untreated WT and HET mice at the dark/light test (D/L), a classical anxiety test that is performed in a large platform that mice can explore almost without stimulation of the whiskers. This test showed an absence of any anxiety phenotype in untreated HET mice (time in the light zone, WT-UNT vs HET-UNT: *t* = 1.090, *p* = 0.2970; unpaired Student’s *t* test; Fig. 2g). In agreement with results obtained in motor testing, the number of transitions between the light and the dark areas was significantly lower in the HET-UNT mice (*t* = 2.585, *p* = 0.0239; Fig. 2h). Furthermore, we analyzed the anxiety-related parameters of the open-field test, which confirmed the absence of any anxiety phenotype also in this test (*U* = 11, *p* = 0.0663, Mann-Whitney test; Fig. 2i). Altogether, these findings strongly support the view that whisker hypersensitivity underlies the preference for the open arms at the EPM observed in both HET female (present study and [37]) and null male *Mecp2*^tm1.1Bird^ mice [31, 42].

### Alterations in parvalbumin-positive cells are rescued by MTZ

**Fig. 3:**
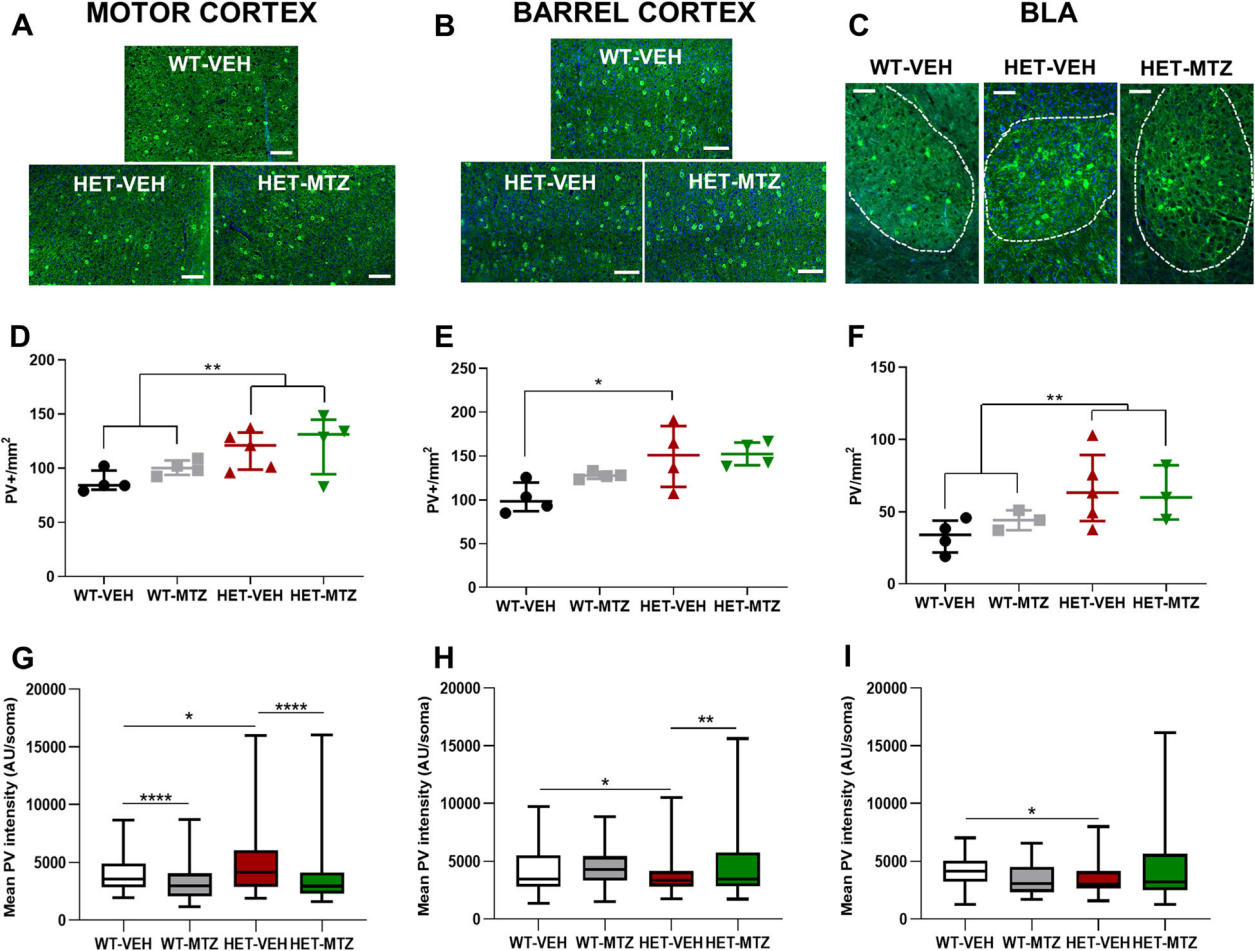
Analysis of the effects of treatment with MTZ (10 mg/kg) on parvalbumin-positive interneurons (PV+ INs) in 6-month-old *Mecp2*^tm1.1Bird^ female mice. **a**–**c** Examples of PV and Hoechst staining of brain sections in the three regions of interest (ROIs) analyzed: the primary motor cortex, the barrel cortex, and the basolateral amygdala (BLA). (scale bar: 50 μm). **d**–**f** Density of PV+ INs in the three ROIs. **g**–**i** Mean intensity of PV+ INs’ somata within the three ROIs. All data are expressed as median ± interquartile range, *n* = 4–5 mice per group; 177–283 neurons per mouse brain (for cortical ROIs) or 38–88 neurons per mouse brain (for BSA). According to results of Saphiro-Wilk test, either one-way ANOVA, followed by Tukey’s post hoc test, or Kruskal-Wallis test, followed by corrected Dunn’s post hoc test was performed. Multiple selected comparisons comprehended: WT-VEH vs WT-MTZ, WT-VEH vs HET-VEH, and HET-VEH vs HET-MTZ. In order to determine an eventual genotype effect, 2-way ANOVA was performed (**d** and **f**). ns: *p* > 0.05 (not shown); *p ≤ 0.05; **p ≤ 0.01; ***p ≤ 0.001

A previous study conducted in 5xFAD transgenic mice, an animal model used to study Alzheimer’s disease, evidenced a preference for the open arms in the EPM similar to the behavior of our adult HET female mice [45]. The authors further demonstrated that 5xFAD mice presented a decreased expression of parvalbumin (PV) in barrel cortex interneurons. Parvalbumin-positive (PV+) cells represent a subpopulation of fast-firing GABAergic cells which accounts for 40% of all interneurons in the rodents’ neocortex [46]. Authors suggested that the reduced levels of PV, which acts as a buffer for free Ca^2+^ rise following action potentials [47], could reflect attenuation of the inhibitory activity of PV+ cells, thus possibly causing whisker hypersensitivity [45]. Notably, altered levels of PV expression in layers II–III of both primary somatosensory and primary motor cortices of *Mecp2*^tm1.1Jae^ male mice have been recently described, suggesting an alteration of the excitation/inhibition balance [48, 49]. We thus hypothesized that the phenotype observed in *Mecp2*^tm1.1Bird^ adult female mice could be linked to alterations in PV+ cells. To test this hypothesis, the cell density and the staining intensity of PV+ cells were measured in three brain areas: primary somatosensory-barrel cortex (S1BF, all layers), primary motor cortex (M1, all layers), and basolateral amygdala (BLA) (Fig. 3; Supplementary Fig. S2). Since in Rett syndrome protein expression is not only modified by cell-autonomous mechanisms but may also be influenced by the non-cell-autonomous environment [50], we decided not to take into account the genotype of each individual cell but rather evaluate the general PV expression in specific brain areas of heterozygous mice. Of note, we have verified the heterozygosis level, and we found that the proportion of Mecp2-KO cells/total cells is on average around 50% in both HET groups (treated and untreated) and therefore the two groups are fully comparable. Whereas neither the general cell density of Hoechst-positive cells nor the thickness of the S1 and M1 cortex was significantly different between groups (Supplementary Fig. S3), the density of PV+ cells in HET mice was significantly increased compared to WT mice in the three analyzed brain areas (M1: *F* = 9.20, *p* = 0.0096; S1BF 11.76, *p* = 0.0050; BLA: *F* = 6.79, *p* = 0.0244; two-way ANOVA). The increased PV+ cell density was not modified by MTZ treatment in any analyzed areas (Fig. 3d–f). Furthermore, the PV immunoreactivity intensity in these neurons was significantly increased in the primary motor cortex (WT-VEH vs HET-VEH: *U* = 14,189, *p* = 0.0049, Mann-Whitney test; Fig. 3g), while it resulted significantly decreased in both barrel cortex (*U* = 24,070, *p* = 0.0403; Fig. 3h) and BLA (*U* = 625.5, *p* = 0.0138; Fig. 3i). Importantly, by comparing HET-VEH and HET-MTZ mice, we observed a complete recovery to normal PV immunoreactivity levels in the motor cortex upon MTZ treatment (*U* = 11,304, *p* < 0.0001; Fig. 3g). Moreover, the low PV immunoreactivity observed in the barrel cortex of HET-VEH mice was significantly increased in HET-MTZ mice, even beyond WT-VEH levels (*U* = 30,430, *p* < 0.0102; Fig. 3h). In contrast, in the BLA, the differences in PV staining in HET-MTZ mice were not statistically significant (*U* = 1909, *p* = 0.2694; Fig. 3i). Taken together, these cytological results are evidence of a structural effect of MTZ on neuronal networks and are in perfect agreement with the observed motor and somatosensory behavioral results.

### Prolonged MTZ treatment prevents from worsening and/or improves RTT symptoms

In addition to the pre-clinical studies, we performed a retrospective analysis on 80 adult female RTT patients (mean age ± SD = 23.1 ± 7.5 years, range = 16–47 years; Table 1) that were admitted to the Hospital “Santa Maria alle Scotte” in Siena during the same time period. In half of these RTT patients (*n* = 40), MTZ was used for treating anxiety and mood disorder with poor sleep quality, according to standard medical indications [51]. Rett clinical severity scale (RCSS) and motor behavioral assessment scale (MBAS) were routinely used as core measures to monitor disease progression by two well-experienced clinicians (JH and CDF), and these two scales were retrospectively analyzed to evaluate MTZ effects on patients.

The treated cohort (MTZ) was compared with a group of 40 untreated (UNT) RTT patients of comparable age (MTZ: age = 23.9 ± 7.9 years, range = 16–47 years vs UNT group 22.3 ± 7.2 years, range = 16–40 years; *p* = 0.354) and disease severity at the initial visit (MTZ treated group RCSS ± SD = 22.6 ± 6.5, range 7–37 vs UNT group 24.7 ± 8.2, range 7–44; *p* = 0.230, Mann-Whitney test for independent samples; Table 1). Also, the global and the area-specific MBAS scores were comparable in the two groups (MTZ-treated group global MBAS ± SD = 58.7 ± 6.5 vs UNT group 56.4 ± 9.0; *p* = 0.596, Mann-Whitney test for independent samples; Table 1). Mean daily and daily/kg body weight doses of MTZ were 11.72 ± 6.96 mg/day (range = 3.75–30.0) and 0.28 ± 0.18 mg/kg body weight/day (range = 0.17–1.07), respectively (Table 2). The mean duration of MTZ treatment was equal to 1.64 ± 1.0 years (range = 0.08–5.0; Table 2). The treatment was well tolerated by 36 patients, while 4 patients were discontinued due to paradoxical anxiety behavior (Table 2). Thus, the NNT (number needed to treat)/NNH (number needed to harm) for this adverse event was estimated to be 10.25 (95% CI 5.026–259.8) with a relative risk of 9.0 (95% CI 0.5004 to 161.87) as compared to the untreated RTT population (z statistic = 1.49; *p* = 0.1361). The observed drug intolerance was unrelated to patients’ age (*p* = 0.5054), clinical stage (*p* = 0.6864), RTT clinical phenotype (typical vs. preserved speech variant; *p* = 0.5533), disease severity (RCSS at baseline, *p* = 0.2307; MBAS at baseline, *p* = 0.4983), or per kg MTZ dosage (*p* = 0.3913) (see Table 3).

**Table 3:** Demographics and relevant features of RTT patients well tolerating MTZ treatment vs patients exhibiting drug intolerance

Variable	Tolerated MTZ	Not tolerated MTZ	*p* value
*N*	36	4	
Age (years)	23.8 ± 8.1	24.7 ± 7.4	0.829
Clinical phenotype			0.553
Typical	33 (91.7%)	4 (100%)	
Preserved speech variant (PSV)	3 (8.3%)	0	
*MECP2* mutation category			0.514
Missense	12 (33.3%)	1 (25%)	
Early truncating	14 (38.9%)	3 (75%)	
Late truncating	9 (25%)	0	
Gene deletions	1 (2.8%)	0	
*MECP2* hotspot mutations			0.269
R133C	2 (5.6%)	0	
R168X	3 (8.3%)	0	
R255X	4 (11.1%)	0	
R270X	1 (2.8%)	0	
R294X	2 (5.6%)	2 (50%)	
R306C	2 (5.6%)	0	
T158M	3 (8.3%)	0	
R106W	0	0	
Other	19 (52.8%)	2 (50%)	
Rett syndrome clinical severity scale (RCSS)	22.9 ± 6.7	19.6 ± 2.5	0.284
Motor behavioral assessment scoring scale (MBAS)	54.7 ± 7.1	51.5 ± 2.4	0.380
I Social-behavioral domain	24.6 ± 7.5	20.5 ± 1.3	0.287
II Orofacial/respiratory domain	15.0 ± 3.7	11.7 ± 1.3	0.091
III Motor and clinical features domain	20.1 ± 2.8	19.2 ± 1.9	0.550
MTZ daily dose (mg/day)	10.9 ± 6.5	13.1 ± 3.7	0.519
MTZ per kg daily dose (mg/kg bw/day)	0.291 ± 0.189	0.297 ± 0.105	0.950
MTZ treatment duration (years)	1.4 [1.1–2.0]	0.3 [0.08–1.2]	0.031

Patient’s diagnosis is indicated as typical or preserved speech variant (PSV). Symptomatic severity was assessed through the Rett syndrome clinical severity scale (RCSS) and motor behavioral assessment scoring scale (MBAS). All patients were females. Data are expressed as mean ± standard deviation or median [inter-quartile range] or *N* (percentages)

**Fig. 4:**
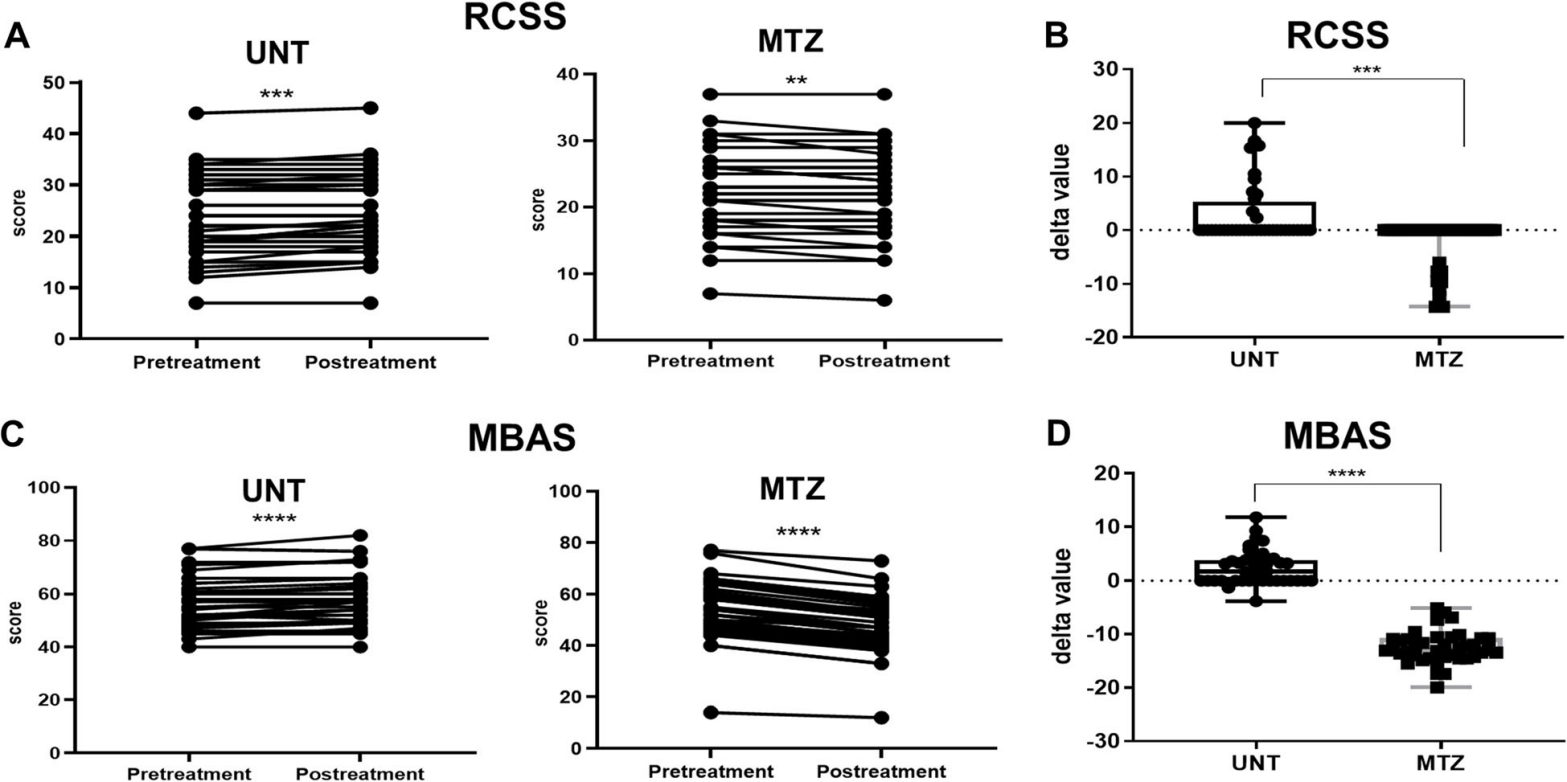
Effects of MTZ treatment on RCSS and MBAS in RTT patients. **a** Rett clinical severity scale (RCSS), scores before and after the treatment (or an equivalent time period when no treatment was present) for each patient are individually represented. **b** Comparison of RCSS delta averages between groups. **c** Motor behavioural assessment scale (MBAS), scores before and after the treatment (or an equivalent time period when no treatment was present) for each patient are individually represented. **d** Comparison of MBAS delta averages between groups. Data are expressed as either paired values (**a**, **c**) or boxplots (**b**, **d**). In all cases, according to results of Shapiro-Wilk test, we used paired Student’s *t* test or Wilcoxon signed rank test for comparisons between pre and post-treatment time points, while we used unpaired Student’s *t* test or Mann-Whitney test to compare averages between treated (*n* = 11) and untreated (*n* = 11) groups. ns: *p* > 0.05 (not shown); *p ≤ 0.05; **p ≤ 0.01; ***p ≤ 0.001

**Fig. 5:**
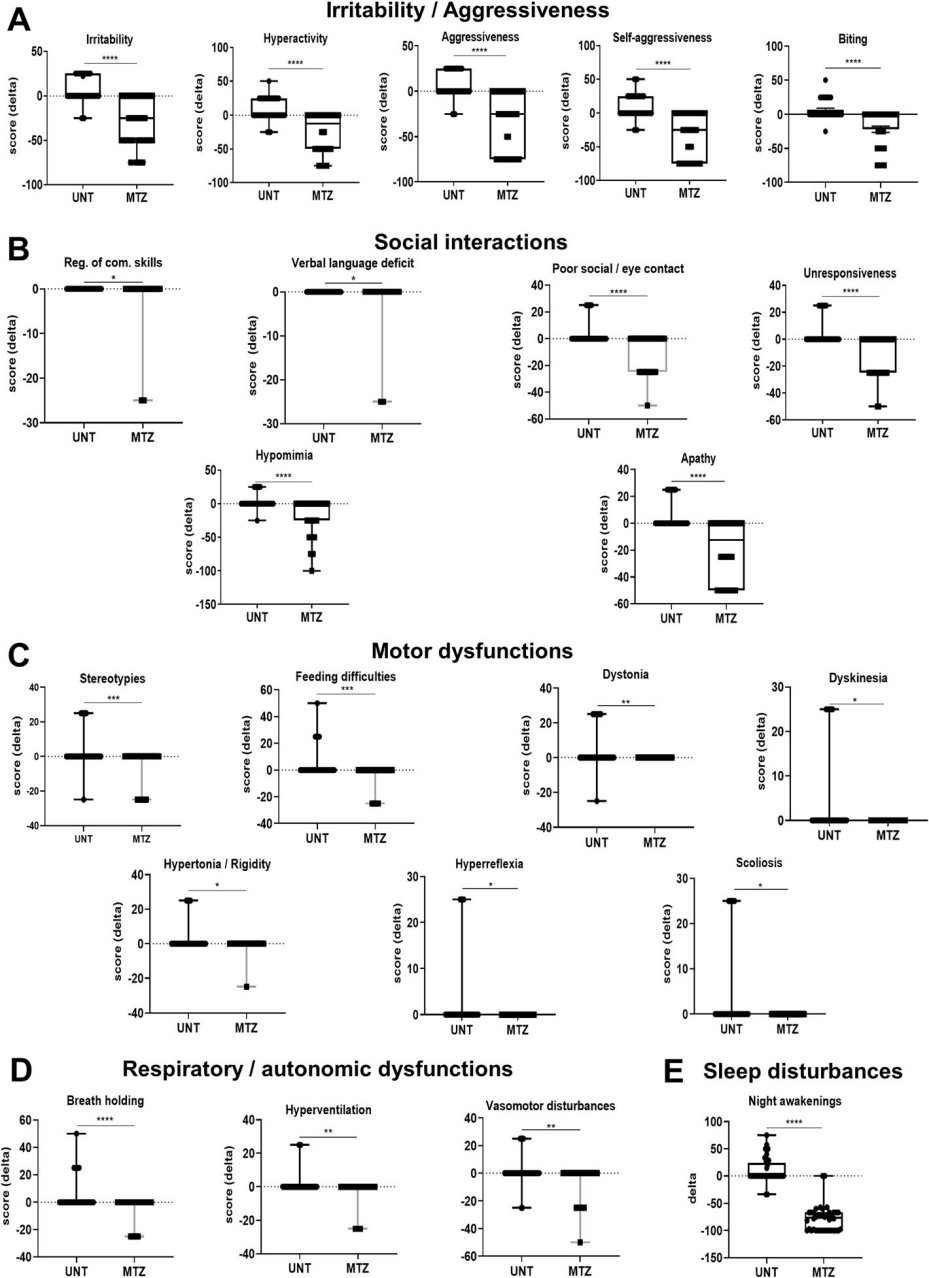
Effects of long MTZ treatment on MBAS subitems and sleep disturbances in RTT patients. **a** Irritability and aggressiveness, **b** social interactions, **c** motor dysfunctions, **d** respiratory and autonomic dysfunctions, and **e** night awakenings. (Complete results are shown in Table 4). All data are expressed as boxplots. In all cases, according to results of Shapiro-Wilk test, we used paired Student’s *t* test or Mann-Whitney test to compare averages between treated (*n* = 11) and untreated (*n* = 11) groups. ns: *p* > 0.05 (not shown); *p ≤ 0.05; **p ≤ 0.01; ***p ≤ 0.001

Given the RTT progressive natural history and the wide inter-individual variability, we evaluated the delta variations (i.e., the difference between values at the end of the observational time period vs values at the initial time point) of both clinical scores and MBAS sub-scores, in order to estimate the possible efficacy of MTZ. UNT patients showed significantly worsened symptoms (i.e., increased values in the values post-treatment vs. pre-treatment) for both total RCSS (*t* = 3.766, *p* = 0.0005, two-tailed *t* test for paired samples; Fig. 4a) and MBAS scores (*t* = 4.759, *p* < 0.0001, two-tailed *t* test for paired samples; Fig. 4c). At the opposite, the MTZ-treated group showed significantly improved clinical scores (i.e., reduced values in the values post-treatment vs. pre-treatment) for both RCSS (*t* = 3.250, *p* = 0.0024 two-tailed *t* test for paired samples; Fig. 4a), and MBAS (*p* < 0.0001, two-tailed Wilcoxon matched-pairs rank test; Fig. 4c), thus indicating improvement of symptoms. In addition, when comparing delta values of both groups, we observed in MTZ-treated patients a highly significant reduction (i.e., clinical improvement) for both RCSS (*p* < 0.0001, Mann-Whitney; Fig. 4b) and MBAS values (*p* < 0.0001, Mann-Whitney; Fig. 4d) with respect to the untreated group. Interestingly, Spearman rank correlation analysis showed that delta changes of MBAS subscores were independent of treatment dosage and duration (analysis not showed). MBAS is a 37-item scale categorized in three main areas: social behavior (M1, 16 items), oro-facial/respiratory (M2, 7 items), and motor/physical signs (M3, 14 items) [52]. Specific results for each MBAS item are shown Table 4 (all results) and in Fig. 5 (statistically significant results, only). Comparing the delta value medians for each MBAS item in the MTZ-treated and UNT patient cohorts, we found statistically significant results in the MTZ-treated group for 22 items in all the three areas of the test with 10/16 items of the M1 social behavior area, 4/7 items of the M2 oro-facial/respiratory area, and 8/14 items of the M3 motor/physical signs area (Table 4). More in detail, we observed that MTZ treatment induced a significant improvement in 5 items of the M1 area, namely, lack of sustained interest/apathy, irritability, hyperactivity, aggressiveness, self-aggressiveness (for all, *p* < 0.001 Mann-Whitney two-tailed test; Table 4), and showed a statistically significant protective effect for the other 17 items, in which MTZ treatment prevented further worsening of the symptoms (Table 4). By a closer look at the type of symptoms described by the 22 MBAS items that resulted significantly modified, we noted that MTZ treatment induced significant effects on clusters of signs that we found useful to group in a slightly different way with respect to the originally described MBAS areas [52]. According to this view, MTZ promoted highly significant improvements in an irritability/aggressiveness cluster of symptoms (Fig. 5a: irritability, hyperactivity, aggressiveness, self-aggressiveness, biting—the last sign did not progress), and prevented from worsening in social interactions (Fig. 5b: regression of communication skills, verbal language deficit, poor social/eye contact, unresponsiveness, hypomimia, lack of sustained interest/apathy—the last shows actually, an improvement); motor dysfunctions (Fig. 5c: hand-stereotypies, feeding difficulties, dystonia, dyskinesia, hypertonia/rigidity, hyperreflexia, scoliosis); and respiratory/autonomic dysfunctions (Fig. 5d: breath holding, hyperventilation, vasomotor disturbances). Finally, we also found that MTZ treatment markedly reduced the number of night awakenings (Fig. 5e, *p* < 0.0001). Taken together, these results are evidence of the positive effects of MTZ in multiple disease domains in adult female RTT patients.

**Table 4:**
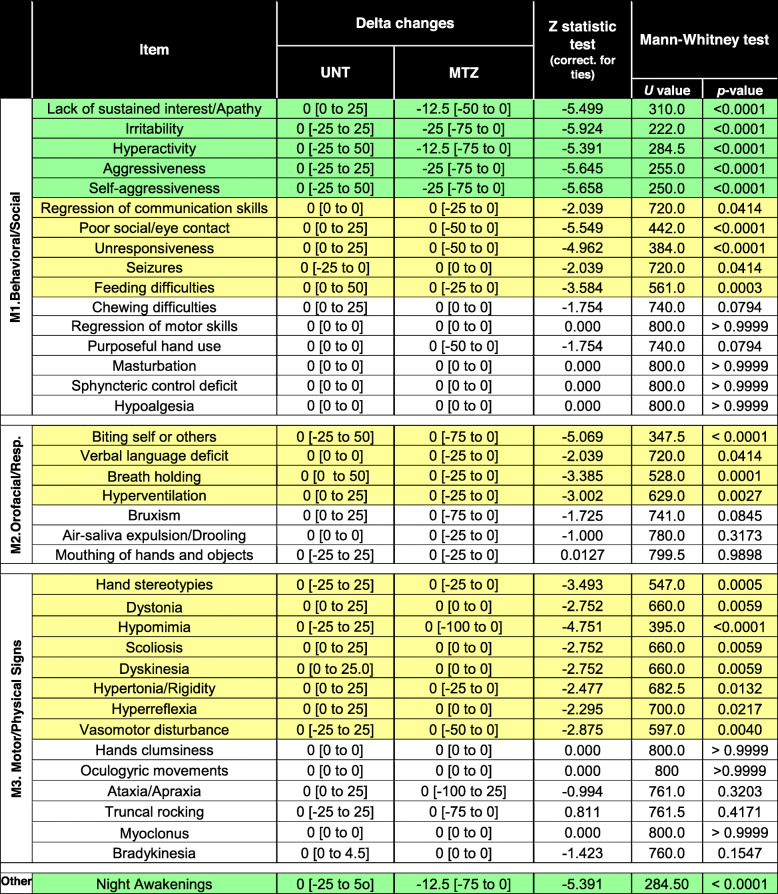
Changes of clinical features from MBAS scoring scale in the untreated (UNT) and mirtazapine-treated (MTZ) RTT patients cohort

Data are expressed as median and 95% CI for the median. In all cases, a two-tailed Mann-Whitney test was performed. Results associated to statistical significance (*p* value < 0.05) are shown in green, while non-significant results are shown unlabeled. Population size, *n* = 40 for each group

## Discussion

In this study, we found interesting parallelisms between MTZ effects in adult female *Mecp2*^tm1.1Bird^ mice and patients. First, apart from four patients showing a paradoxical anxiety reaction, we did not observe any significant side effects of MTZ long-term treatment in neither *Mecp2*^tm1.1Bird^ mice (30 days) nor RTT patients (up to 5 years). This is a key point in terms of the future usability of this drug in RTT patients, as a high safety profile is a mandatory requirement for any chronic pharmacological treatment. Secondly, MTZ fully rescued an avoidance behavior observed in HET mice when tested in the elevated plus maze, likely reflecting a sensory hypersensitivity of whiskers associated to a reduced PV expression in the barrel cortex. In rodents, whiskers’ hyperstimulation is associated with irritability and aggressiveness, and MTZ significantly improved irritability, aggressiveness, self-aggressiveness, hyperactivity, and biting in RTT patients. Thirdly, MTZ prevented deterioration of motor skills in *Mecp2*^tm1.1Bird^ mice and RTT patients. Indeed, by using two motor tests (horizontal bar and dowel test), which had never been used before in *Mecp2*^tm1.1Bird^ female mice, we showed a full rescue of motor skills and motor learning deficits by MTZ. In parallel, in adult RTT patients, MTZ prevented worsening of hand-stereotypies, feeding difficulties, dystonia, dyskinesia, hypertonia/rigidity, hyperreflexia, and scoliosis. Finally, while the general health scoring was unmodified in HET mice, we reported that in adult RTT patients, MTZ treatment significantly improved general health (evaluated through the RCSS) as well as night-awakenings, and behavioral features regarding social interactions assessed through the MBAS scale, such as regression of communication skills, verbal language deficit, poor social/eye contact, unresponsiveness, hypomimia, and lack of sustained interest/apathy. Furthermore, although we could not carry out similar studies in mice, we found in treated patients a significant protective effect of MTZ on respiratory/autonomic dysfunctions including breath holding, hyperventilation, and vasomotor disturbances. Considering the limited plasticity present in both adult mice and adult humans, it is remarkable that MTZ could decelerate or even improve the RTT and RTT-like phenotypes.

The tests included in our behavioral battery were selected according to previous studies [37, 42] and the order in which we administered the different tests to the mice was based on considerations reported in [53]. We added two new tests that were never used before in assessing motor deficits in animal models of the Rett syndrome, namely the horizontal bar and the dowel tests. The rationale for introducing these new tests is that, since they are almost pure motor tests in which the emotional state of the mouse is practically irrelevant, they were the only tests which could be repeated without introducing a confounding bias. In contrast, all other tests included in our study are subjected to the emotional state of the animal and therefore common practice in behavioral testing on mice is to administer these tests only once in the behavioral test battery. Because of the lack of emotional content, both horizontal bar and the dowel tests could be repeated before, in the middle, and at the end of the treatment time period. This experimental setting allowed us to monitor the motor learning curve of the different mouse groups during the observation time. Of note, WT mice did not show any improvement (or learning) in the dowel test through the treatment period because all of them showed an optimal performance already at the first time they underwent the test, i.e., they did not fall from the wooden dowel for the entire duration of the test (120 s). In contrast, considering that most mice of the group of HET-MTZ showed a very bad performance at the pre-treatment time point, their progressive improvement at the intermediate and then at the end of the treatment compared to the pre-treatment likely reflects a motor learning that was taking place during repeated testing.

In terms of age and genetics, the *Mecp2*^tm1.1Bird^ female mice we used were optimal for comparisons with the adult RTT patients investigated. According to the criteria reviewed in [54], we calculated that the age of HET female mice used in our study (6 months) was equivalent to approximately 25 human years, which is perfectly comparable with the mean age of patients included in the retrospective study (23.1 ± 7.5 years). Moreover, just like female RTT patients, HET female mice present a variable dosage of the *Mecp2* gene, depending on the mosaicism pattern created by the random inactivation of one X chromosome [44]. The heterozygosity in our mouse colony is on average around 50%, meaning that half of the cells express the *Mecp2* wild-type allele, with a range comprised between 30 and 70% (JFG and ET unpublished observations). Despite all its advantages as an animal model to study RTT, *Mecp2*^tm1.1Bird^ female mice show limitations as well. In particular, the high weight gain, common to all HET female mice starting from the third month of life but present only in a low percentage of RTT patients (around 8–10%) obviously impairs the performance in motor tests. This may explain the limited improvement in motor tests, even if MTZ induced a full recovery of normal PV expression in the primary motor cortex. Another limitation regards the fact that social behavior deficits are not evident in adult HET female mice [37, 42] and, therefore, we could not compare this disease dimension, which is relevant in RTT people. Finally, the positive effect of MTZ on aggressiveness observed in patients could not be compared with an analogous behavior in female HET mice. Aggression in mice is usually measured using the resident–intruder test, which evaluates territorial behavior in male rodents [55], while female mice do not display aggressive behavior towards strangers [20, 56]. Using the resident–intruder test, enhanced aggressive behavior has been observed in male conditional knock-out mice lacking *Mecp2* within serotonergic cells [21], but no increased aggressiveness was found in *Mecp2*^308/Y^ male mice [20, 57], although they display enhanced avoidance behaviors [58].

In this study, we propose that *Mecp2*^tm1.1Bird^ mice’s aberrant open arm preference in the EPM is due to sensory hypersensitivity. As previously described in RTT literature [37, 42], we confirmed that female HET mice spend more time exploring the open arms compared to WT littermates. Furthermore, we demonstrate that MTZ can completely normalize the altered behavior at the EPM and that clipping of whiskers in HET mice produced a fully equivalent rescue. This finding strongly suggests that the specific phenotype of HET mice in the EPM is actually not related to abnormal anxiety levels but rather to an avoidance of the closed arms, possibly due to the hypersensitivity of the whiskers. The fact that both open-field and light-dark exploration tests did not show any anxiety-related phenotype in HET mice further strengthens our hypothesis. Mechanical hypersensitivity is an emerging finding in RTT research. Two recent studies showed marked skin hyperinnervation [59], and mechanical hypersensitivity in one male rat model proposed to study RTT, and somatosensory and viscerosensory alteration in female rats of the same model [59, 60]. In striking agreement, another study demonstrated increased sensory fiber innervation in skin biopsies from RTT patients [61]. In RTT people, somatosensory disturbances may range from elevated pain threshold or heat insensitivity [62] to hypersensitivity [63, 64]. Notably, a tactile hypersensitivity on skin and hair has often been noted during routine examinations of patients during our clinical study, likely contributing to increased irritability observed in adult RTT patients (JH and CDF unpublished observations). Accordingly, here we put forward the idea that partial or complete restoration of normal responses to somatosensory stimuli could be one positive outcome of MTZ treatment in RTT.

Regarding the possible mechanism underlying the rescue of the aberrant behavior in the EPM, our histological analyses shed some light on it. Although the overall number of PV+ neurons was slightly increased in both motor and somatosensory cortices, we found that expression of PV was significantly downregulated in the barrel cortex of HET-VEH mice. A previous study showed that altered PV expression correlates with both a decrease in the number of glutamatergic afferents and an increase in GABAergic afferents to PV+ cells, likely leading to a depression of the electrical activity in these cells [49]. Of note, PV+ neurons have been directly linked to the generation of gamma oscillations that modulate afferent information in the barrel cortex [65]. Hence, we propose that in HET-VEH the internal inhibition in the barrel cortex might be decreased, resulting in an upregulated processing into the somatosensory pathway originating in the whiskers. Under these circumstances, whiskers’ sensory stimuli perceived as normal by WT mice are disturbing for HET mice, and as a consequence, HET mice stay longer in the open arms. Within this paradigm, MTZ could have normalized the processing of somatosensory stimuli from whiskers through the rescue of the normal internal inhibition in the barrel cortex, as indicated by the restored levels of PV in HET-MTZ mice. Interestingly, the bottle-brush test, a behavioral test based on a robust stimulation of whiskers commonly used to evaluate irritability in rodents, generates aggressive and defensive behavior [66, 67]. Although human behavior in this domain is much more complex as compared to mice, results obtained in experimental animals at the EPM may be assimilated to the irritability and aggressiveness behaviors which were significantly improved in MTZ-treated patients.

In this study, we have shown the MBAS patients’ data organized according to the classical M1-M3 areas that were originally described in the literature [52]. However, based on a clear affinity between symptoms such as “self-aggressiveness” and “aggressiveness” of the area M1 with “biting self or others” of the M2 area, we propose here an original aggregation of the results into four clusters of improvement/protection effects induced by mirtazapine, namely, irritability/aggressiveness, social interactions (and probably, also attention), motor dysfunctions, and respiratory/autonomic dysfunctions. We believe that this novel subdivision may provide a better basis for further functional studies on the mechanism of action of mirtazapine.

Although the reported effect of MTZ on sleep awakening became known from questionnaires completed by parents or caregivers, it appears to be relevant in the management of disease in terms of quality of sleep and quality of life for the patients and their families. In previous studies, sleep problems have been largely investigated in RTT as a relevant disease symptom by using questionnaires (Young D, 2007), polysomnographic analysis [68], or electroencephalogram (EEG) spectral analysis [69]. In particular, variations in sleep problems were shown to be deeply linked to other RTT clinical features, with a high prevalence according to the mutation type. In particular, sleep problems were least likely to be reported in those RTT patients bearing a *MECP2* gene with C terminal deletions, while night-time laughing was commonest in those with a large deletion and daytime napping was commonly reported in cases with p.R270X, p.R255X, and p.T158M mutations [70]. However, our data show no apparent correlation of sleep disturbances with any specific mutations or mutation type. Sleep quality and respiratory problems may be connected because previous polysomnographic studies in Rett syndrome showed a ventilatory impairment linked to altered respiratory parameters and low mean oxygen desaturation percentage during sleep [68]. Interestingly, in our retrospective case series, we found that treated patients also showed a small, but statistically significant, improvement in hypoventilation ad breath-holding.

A final, important consideration regards the four patients with Rett syndrome who presented paroxysmal anxiety levels and were discontinued after a short time treatment with MTZ. Analysis of their clinical characteristics and genotype did not lead to any conclusive indications of an existing correlation between specific mutations or mutation categories and these adverse events. In fact, one patient had the common missense mutation T158M, one had the Y141X early truncating mutation while the other two were bearing the frequent R294X early truncating mutation. However, another two patients bearing the same R294X mutation could be treated for 2 years reaching the maximal dose of 30 mg/day without any adverse effect. Thus, further investigation is needed to explore the genotype-phenotype relationship for this adverse event.

## Conclusions

The finding that MTZ treatment can rescue a large number of abnormal behavioral features in adult RTT patients is a breakthrough in the field. A recent large observational study on the natural history of RTT has identified behavioral problems as a key emerging issue in RTT [71]. Interestingly, mood and behavioral problems are considered major issues, together with sleeping disturbances, seizures, and breathing problems, in terms of impact on the quality of life of families taking care of RTT girls [72]. Besides the efficacy in regulating sleep and mood disturbances, which was the initial reason for prescription in adult RTT patients, it is noteworthy that MTZ treatment could improve or slow down several of the behavioral abnormalities listed into the RTT natural history study. Taken together, our findings support the potential of MTZ for improving the quality of life of RTT patients and their families.

## Supplementary information

**Additional file 1.** Supplementary Figures

## Data Availability

The datasets used and/or analyzed during the current study are available from the corresponding author on reasonable request.

## Abbreviations

BLA: Baso-lateral amygdala
D/L: Dark/light test
DNA: Deoxyribonucleic acid;
EDTA: Ethylenediaminetetraacetic acid
EPM: Elevated plus maze
GABA: Gamma-aminobutyric acid
HET: Heterozygous-*Mecp2* female mice
MBAS: Motor behavioral assessment scale
*MECP2*: Methyl-CpG binding protein 2
MTZ: Mirtazapine
PCR: Polymerase chain reaction
PSV: Preserved speech variant
RCSS: Rett clinical severity scale
Rpm: Revolutions per minute
RT: Room temperature
RTT: Rett syndrome
SDS: Sodium-dodecyl-sulphate
Tris: Tris(hydroxymethyl)aminomethane
UNT: Untreated female mice
UNTw^-^: Untreated female mice without whiskers
VEH: Vehicle
WT: wild type-*Mecp2* female mice;
XCI: X-chromosome inactivation;
