# Supplementary material for: Protective role of mirtazapine in adult female Mecp2+/− mice and patients with Rett syndrome
Source: J Neurodev Disord. 2020 Sep 28;12:26. doi: 10.1186/s11689-020-09328-z (PMC7523042; doi:10.1186/s11689-020-09328-z)
Supplement: Supplementary file 1 — Additional file 1. Supplementary Figures [file 11689_2020_9328_MOESM1_ESM.docx]

**Supplementary Figure S1**

**
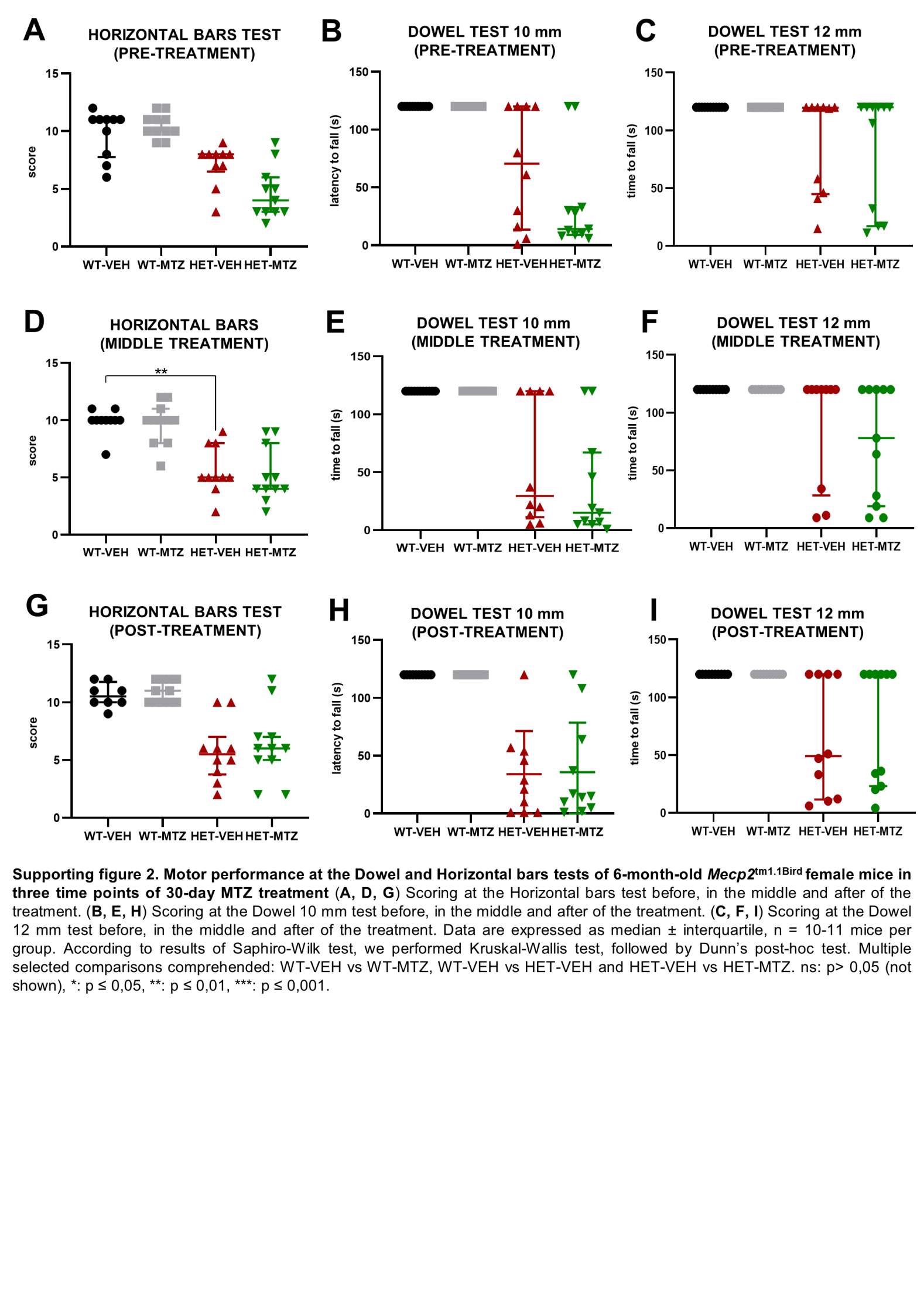
**

**Supplementary Figure S2**

**
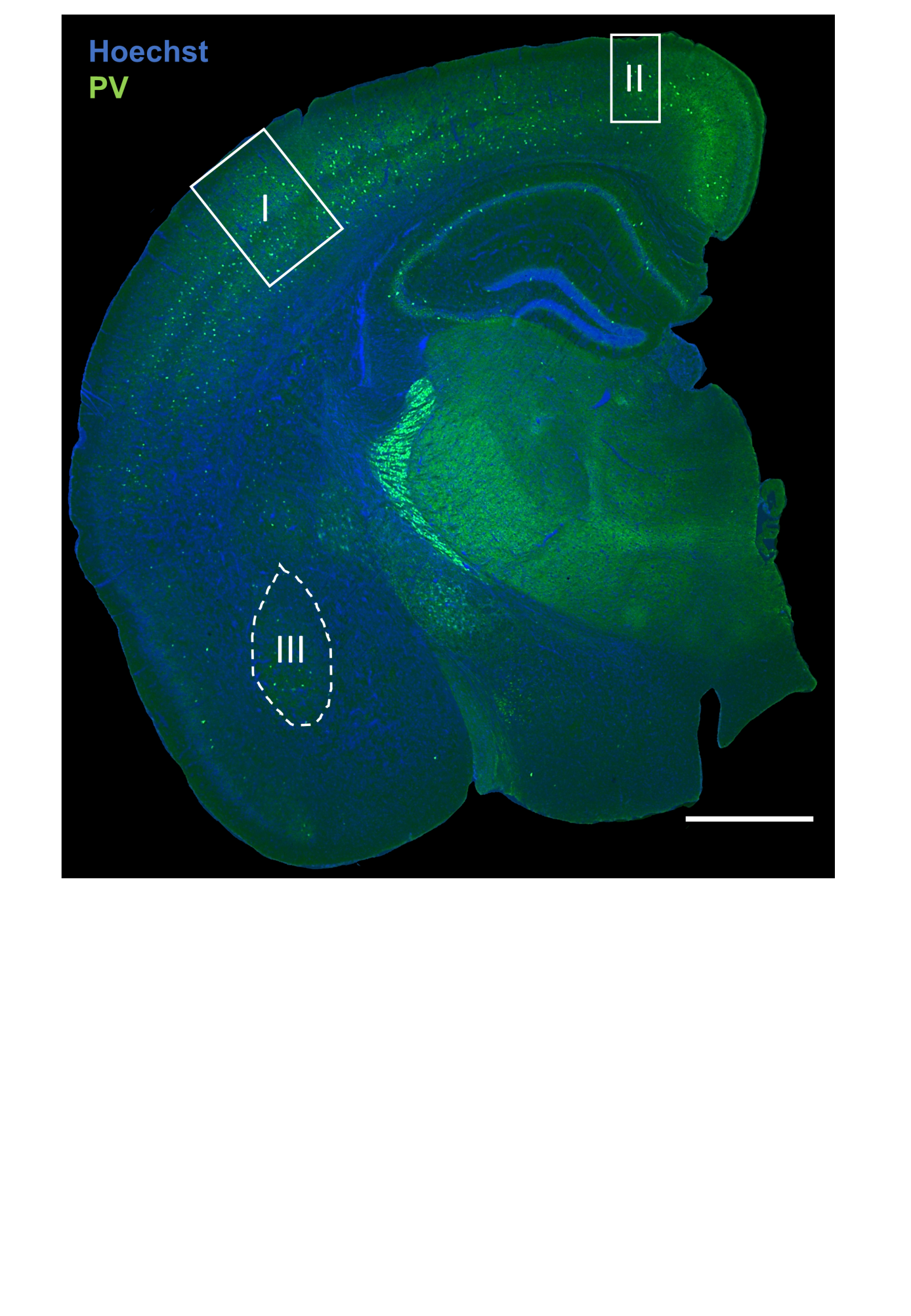
 Supplementary figure S2. Regions of interest (ROIs) analyzed in 6-month-old *Mecp2*^tm1.1Bird^ after treatment with MTZ (10 mg/kg) and behavioural testing (see Fig. 1).** In the picture, an example of PV (green) and Hoechst (blue) staining in a 20-μm brain section from a *Mecp2*^tm1.1Bird^ wild-type mouse treated with VEH is shown. Analyzed ROIs are also indicated: primary somatosensory-barrel cortex (I), primary motor cortex (II) and basolateral amygdala (III). Scale bar: 1000 μm

**Supplementary Figure S3**

**
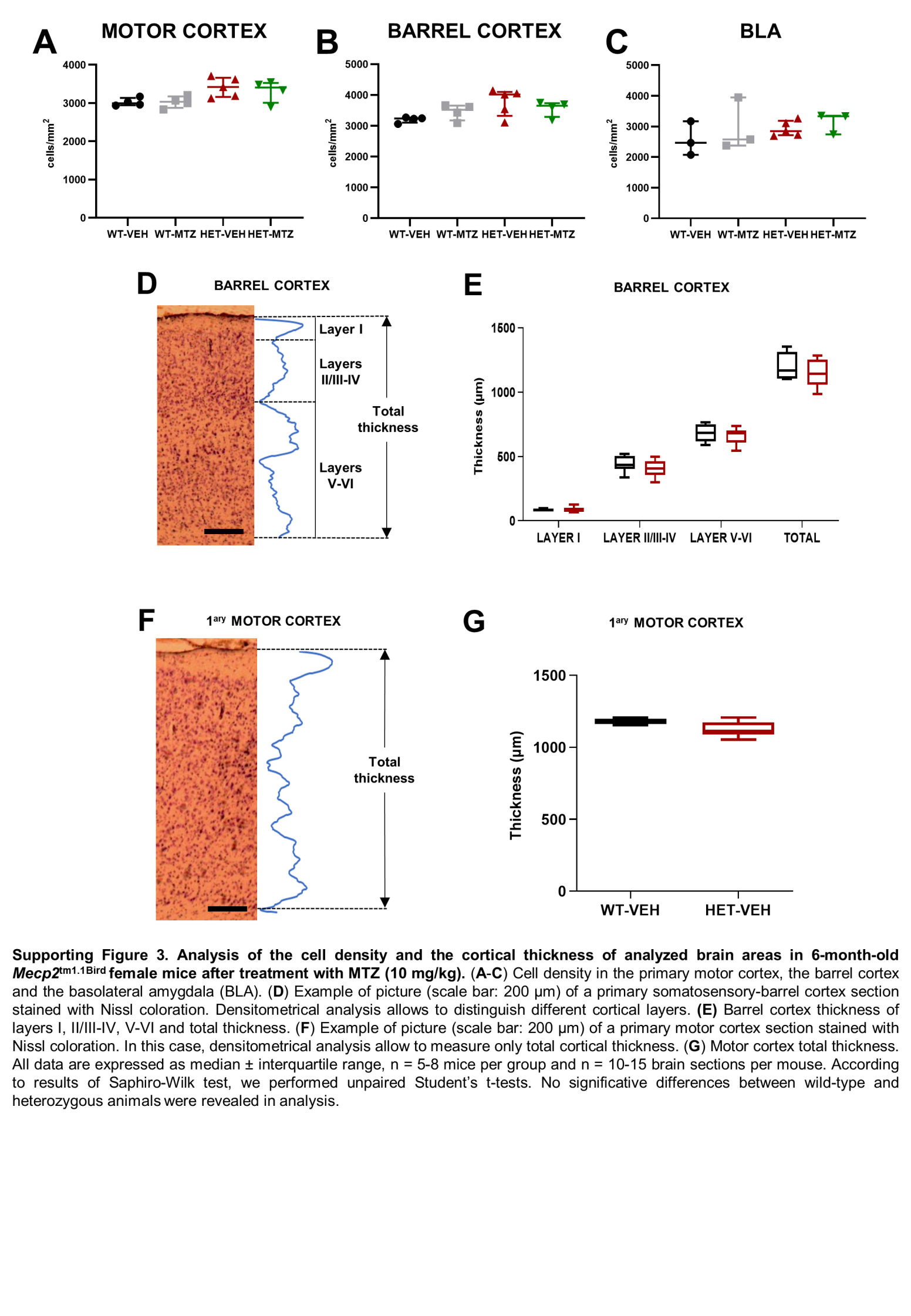
**
